# Comparative Seeds Storage Transcriptome Analysis of *Astronium fraxinifolium* Schott, a Threatened Tree Species from Brazil

**DOI:** 10.3390/ijms232213852

**Published:** 2022-11-10

**Authors:** Leonel Gonçalves Pereira Neto, Bruno Cesar Rossini, Celso Luis Marino, Peter E. Toorop, Edvaldo Aparecido Amaral Silva

**Affiliations:** 1 Embrapa Recursos Genéticos e Biotecnologia (Cenargen), Brasilia 70770-917, Brazil; 2 Biotechnology Institute, São Paulo State University “Júlio de Mesquita Filho”, Botucatu 18607-440, Brazil; 3 Departament of Biological and Chemical Sciences, Biosciences Institute, São Paulo State University “Júlio de Mesquita Filho”, Botucatu 18618-689, Brazil; 4 Department of Comparative Plant and Fungal Biology, Royal Botanic Gardens, Kew, Wakehurst Place, Ardingly, West Sussex RH17 6TN, UK; 5 Departamento de Produção Vegetal, Faculdade de Ciências Agronômicas, Universidade Estadual Paulista, Botucatu 18610-034, Brazil

**Keywords:** seed longevity, differentially expressed genes, transcription factors

## Abstract

*Astronium fraxinifolium* Schott (Anacardiaceae), also known as a ‘gonçalo-alves’, is a tree of the American tropics, with distribution in Mexico, part of Central America, Argentina, Bolivia, Brazil and Paraguay. In Brazil it is an endangered species that occurs in the Cerrado, Caatinga and in the Amazon biomes. In support of ex situ conservation, this work aimed to study two accessions with different longevity (p50) of *A. fraxinifolium* collected from two different geographic regions, and to evaluate the transcriptome during aging of the seeds in order to identify genes related to seed longevity. Artificial ageing was performed at a constant temperature of 45 °C and 60% relative humidity. RNA was extracted from 100 embryonic axes exposed to control and aging conditions for 21 days. The transcriptome analysis revealed differentially expressed genes such as Late Embryogenesis Abundant (*LEA*) genes, genes involved in the photosystem, glycine rich protein (*GRP*) genes, and several transcription factors associated with embryo development and ubiquitin-conjugating enzymes. Thus, these results contribute to understanding which genes play a role in seed ageing, and may serve as a basis for future functional characterization of the seed aging process in *A. fraxinifolium*.

## 1. Introduction

Seed persistence in natural habitats depends on the physical and physiological characteristics of the seed, which varies among species and populations [1]. The effects of climate change can affect flowering in forests and, therefore, there is a need to also evaluate seeds for this type of stress [2]. The continuous human exploitation causes deforestation and necessitates conservation to mitigate the loss of plant diversity. The maintenance of germplasm in seed banks serves as a valuable effort, contributing to that conservation goal [3]. Seed quality is strongly influenced by the inevitable process of aging, including during long-term storage. Currently, the process of aging tests in germplasm banks evaluates the viability of the seeds through a germination test; however, this kind of test does not allow for the protection of the progress of events that underlie the deterioration, indicating only the final stages of the process [4]. This process of monitoring the viability of specimens kept in germplasm banks is a routine practice of the banks, and refers to the evaluation of the physiological quality of the seeds during storage [5]. Thus, alternatives to the evaluation of seed deterioration in seed banks are studied in order to quickly identify their viability [6,7,8,9]. Seeds of different species and cultivars, preserved in germplasm banks under the same conditions of temperature and humidity, present different responses regarding the loss of viability [1]. This longevity is affected by several factors such as the chemical composition of the seed, maturation stage, initial viability, humidity, temperature and degree of infection by microorganisms and insects [10,11,12,13]. One of the main challenges to keep seed viability in germplasm banks is to predict when the accessions should be regenerated and to detect the initial stages of seed deterioration without consuming the samples in repetitive trials to evaluate the viability in the monitoring process [4].

The process of seed deterioration is considered as the reduction of physiological quality, related to a complex of changes that occur over time and that causes damage to the systems and vital functions, resulting in the decrease or loss of the capacity and performance of the seed [14]. Initial stages of deterioration should ideally be detected early during the storage of seeds in order to elucidate the behavior differences between the species with seeds of different qualities during the conservation period. Several events can occur in the seed before loss of total viability, such as the disruption of the membrane system, the decrease in the activity of respiratory enzymes, the reduction in the efficiency of DNA repair mechanisms, among others [15,16,17]. In addition, seed deterioration during storage leads to an accumulation of damage to the cell structure, DNA, RNA, proteins and lipids with fragmentation of molecules which are stored in the dried seed [18,19]. Thus, biochemical, physiological and molecular tests can be used as useful tools to indicate possible damages caused to the seeds, allowing a better management of germplasm collections. Molecular studies for evaluating seed deterioration have been increasingly used [8,9,20,21]. The use of tests complementary to those traditionally employed in the evaluation of the quality of the seeds may add information that will allow for greater understanding about the aging process and the deterioration of seeds and, consequently, considerably improve the management of collections in germplasm banks. Therefore, with the early identification of seed deterioration based on molecular techniques, for example, and with a reduced input of samples, it can significantly contribute to the maintenance of stocks, implying less need for new collections in nature. The study of gene expression among seeds with natural or accelerated aging shows similar results at transcriptional levels, with differences for small numbers of genes between varieties that are sensitive or more tolerant to ageing [22]. Thus, several studies have analyzed the gene expression profile with a focus on development in order to identify possible genes responsible for the aging process in order to detect the beginning of the decline in seed viability [9,23,24]. Furthermore, the amount of mRNA present between short-lived and long-lived seed accessions, in addition to different associated processes, such as the presence of more heat-shock proteins in long-lived seed accessions, demonstrates that the interactions between genes are complex and determined by many factors [13].

*Astronium fraxinifolium* Schott (Anacardiaceae) is a tree of the American tropics that is found in Mexico, parts of Central America, Argentina, Bolivia, Brazil and Paraguay [25,26]. In Brazil it occurs in the Brazilian savannah, Caatinga and in the Amazon biomes. The species is known as ‘gonçalo-alves’ and has great economic importance due to the quality of its wood [26,27]. Thus, due to overexploitation, its current distribution is limited to small forest fragments and the margins of highways, and as a result the species is considered to be threatened with extinction [28,29]. *Astronium fraxinifolium* was also studied in the International Space Station (ISS) by the Brazilian astronaut Coronel Marcos César Pontes in an experiment with seed germination in micro gravity [30].

For native species, hardly any studies are available on the molecular processes involved in seed deterioration during storage, mainly related to long-term conservation, which makes it difficult to establish adequate predictive tools for the conservation of these species. Therefore, the present work aims to use the different seed longevity of *A. fraxinifolium* Schott accessions collected from two different geographic regions, evaluate the transcriptome during the aging of these seeds, and suggest possible markers associated with the aging process.

## 2. Results

### 2.1. Effects on Physiological Indexes during A. fraxinifolium Seed Treatment

**Figure 1:**
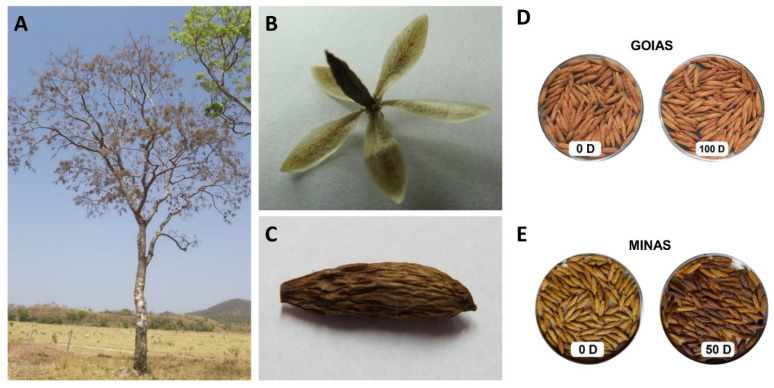
Morphological details of species, seeds and fruits of *A. fraxinifolium* (**A**) *A. fraxinifolium* tree. Tree without leaves and only fruits present. The tree has a medium arboreal size, with a height of 8 to 12 m, a cylindrical and straight trunk, 60 to 80 cm in diameter at breast height (DBH) and with whitish bark; (**B**,**C**) Fruit of *A. fraxinifolium* adhered to the calyx. The fruit is a pseudo-samara, with a uniseriate exocarp, suberified and attached to the mesocarp; (**D**) Seeds from accession GOIAS, submitted to the accelerated aging test at 60% RH with zero days of aging and 100 days of accelerated aging; (**E**) Seeds from accession MINAS, submitted to the accelerated aging test at 60% RH with zero days of aging and 50 days of accelerated aging.

The accelerated aging process revealed great differences between the two accessions (Table 1). Germination was lower in accession MINAS than in GOIAS, with browning of seeds observed (Figure 1).

### 2.2. Sequencing Transcriptome Profile

In order to obtain a set of genes involved in seed longevity of *A. fraxinifolium*, high throughput RNA sequencing was performed with the two accessions using GOIAS and MINAS controls with 98 and 93% of germination, respectively; and ageing induced GOIAS and MINAS with 97 and 81% of germination, respectively. Filtered reads totaled more than 90 million per treatment, with 124,528 transcripts assembled for all sequenced reads from samples. Sequencing reads were deposited at the National Center for Biotechnology Information (NCBI) under SRA BioProject accession number PRJNA881610. These filtered reads were mapped against assembled transcriptome reads with more than 85% successfully mapped reads (Table 2).

### 2.3. Comparison of Seed Longevity and Identification of Differentially Expressed Genes

**Figure 2:**
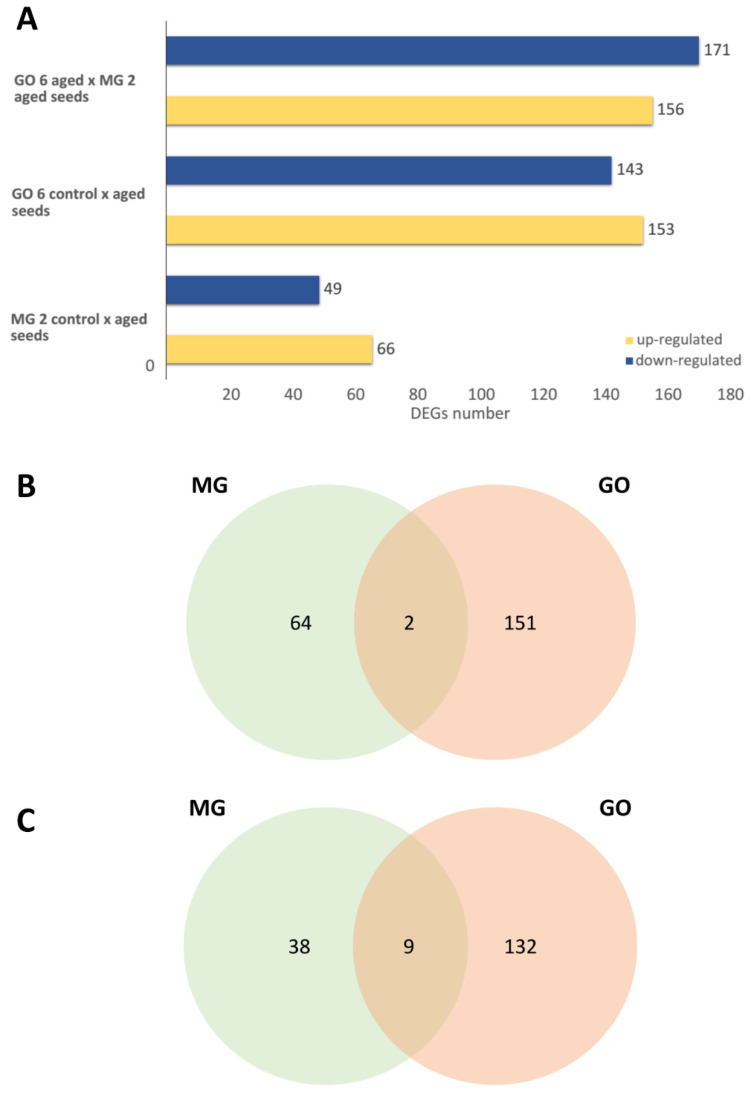
Statistical analysis of differentially expressed genes (DEGs) from *A. fraxinifolium* seed germination treatments. (**A**) Number of differentially expressed genes (DEG) in *A. fraxinifolium* seeds in comparison between seed treatments with and without aging (log2fold > 2, Padj ≤ 0.05). (**B**) Venn diagram of DEG upregulated between treatments of *A. fraxinifolium* seeds from MINAS (MG) × GOIAS (GO) accessions with aged seeds. (**C**) Venn diagram of downregulated DEG between treatments of seeds of *A. fraxinifolium* from accessions MINAS and GOIAS with aged seeds.

The differentially expressed genes analysis identified 296 genes for GOIAS aged vs. control seeds, 115 genes for MINAS aged vs. control seeds, and 327 genes against GOIAS aged vs. MINAS aged seeds (Figure 2). The comparison between treatments GOIAS control × MINAS control seeds showed no significant differentially expressed genes. The complete list of DEGs can be found in Supplementary Table S1.

Among the DEGs identified as up-regulated in both treatments with aged seeds, two genes are common. Their functions are related to *ABC transporter B family member 26* (c31142_g1_i6) and *Myosin-15* (c29015_g2_i10). On the other hand, down-regulated DEGs are related to regulation, endocytosis and development (Supplementary Table S2).

**Figure 3:**
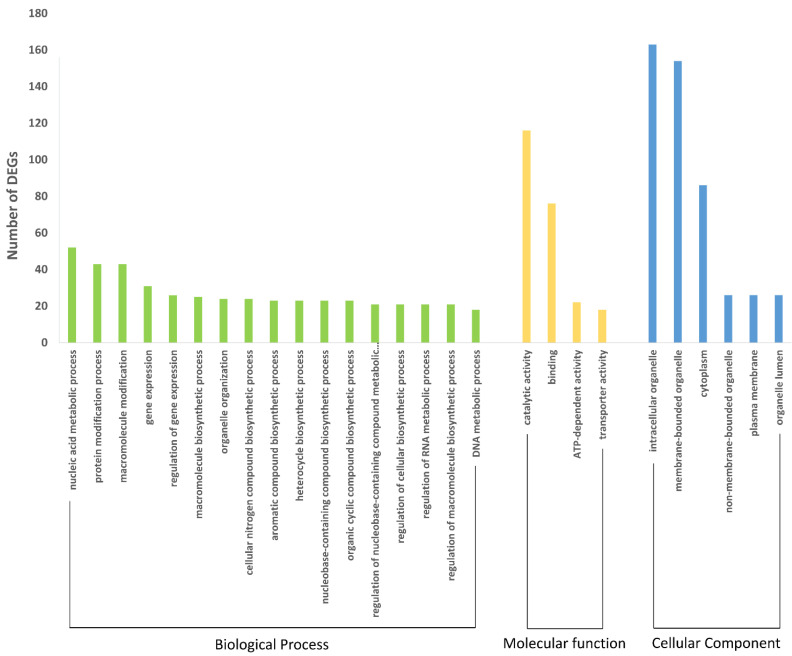
Gene ontology terms identified for DEGs in both treatments. Each color represent one main category: green for biological process, yellow for molecular function, and blue for cellular components. In the x axis the GO terms are represented.

**Figure 4:**
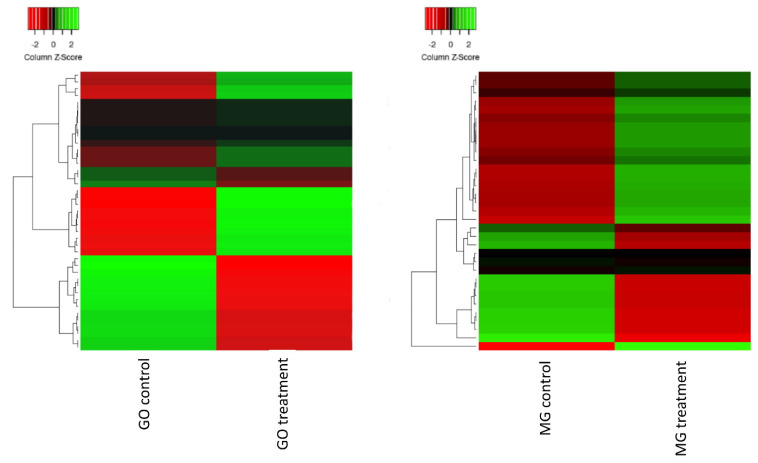
Heatmap of selected genes possibly involved with the aging/longevity process exhibited in Table 3. Red color indicates highly expressed genes (up regulated), and green represents the down-regulated genes. The green to red color transition reflects the values of an FPKM normalized log2-transformed counts.

In order to identify the functions associated with differentially expressed genes, we performed a GO analysis. Thus, of all genes identified between treatments, we annotated and categorized them in three classes: biological process, molecular function and cellular component. Of these, the most representative for biological process were the nucleic acid metabolic process (11.2%), the protein modification process (9.3%) and macromolecule modification (9.3%); for molecular function catalytic activity (50%), binding (32.7%) and ATP-dependent activity (9.4%) were most abundant; and finally, for cellular component intracellular organelle (33.8%), membrane-bounded organelle (32%) and cytoplasm (17.8%) were observed most frequently (Figure 3). Considering the comparisons between GOIAS control vs. induced aged seeds, the main GO terms in both up and down regulated genes are related to regulation of transcription, response to stimulus, transport and protein phosphorylation. When considering MINAS control vs. induced aged seeds, the main GO terms from upregulated genes are related to DNA repair, chromatin organization, regulation of transcription, and protein ubiquitination. For the down-regulated genes, the main GO terms are related to transmembrane transport, cell differentiation, phosphorylation, and signal transduction (Supplementary Table S1). From these, we selected some genes possibly directly related to the aging process in each treatment (Table 3). A heatmap of these selected genes possibly involved with the aging/longevity process is presented in Figure 4.

**Figure 5:**
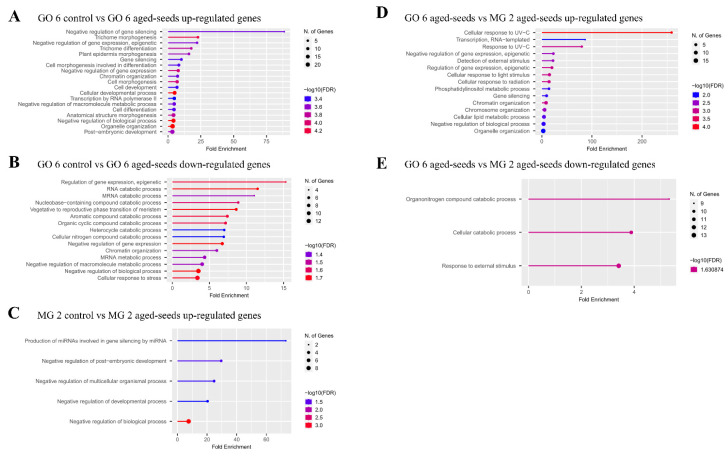
Gene Ontology (GO) enrichment analysis. (**A**,**B**) Enrichment analysis of DEGs that were up and down expressed in the comparison of GOIAS control vs. GOIAS aged-seeds. (**C**) Enrichment analysis of DEGs that were up expressed in the comparison of MINAS control vs. MINAS aged-seeds. (**D**,**E**) Enrichment analysis of DEGs that were up and down expressed in the comparison of GOIAS aged-seeds vs. MINAS aged-seeds.

Gene Ontology enrichment analysis revealed that most genes are mainly related with the control of gene expression in the GOIAS control vs. GOIAS aged-seeds comparison (Figure 5). When considering MINAS control vs. MINAS aged-seeds, most results are related to the production of miRNA involved in gene silencing and the negative regulation of development, with no enriched pathways found for down-regulated genes. Finally, when comparing GOIAS aged-seeds vs. MINAS aged-seeds, the enriched pathways are mainly related to the response to external stimulus, such as UV-light (Figure 4; Supplementary Tables S3–S5).

### 2.4. Identification of Transcription Factors and Related Transcription-Mediated Complex

Based on annotation of the DEGs, transcription factors and mediators of RNA polymerase were identified. In GOIAS control vs. GOIAS aged-seeds, six transcripts encoded for putative mediators/co-activation of RNA polymerase II, four for transcription factors and three for transcription activators/adapters. In the MINAS control vs. MINAS aged-seeds comparison, one transcript encodes a transcription factor and one other a transcription initiation factor. Finally, in GOIAS aged-seeds vs. MINAS aged-seeds comparison, two transcription factors, one transcription initiation factor and one mediator of RNA polymerase were found, but also one co-repressor (Table 4).

## 3. Discussion

When stored for long periods, seeds eventually lose their germination capacity, caused by loss of viability. This affects not only commercial operations but also ex situ seed banks for species conservation representing remaining in situ populations. The ex situ maintenance of viable seeds covering wide genetic variability is an extremely important process for the genetic conservation of species [31]. In the case of long-lived tree species, such as *A. fraxinifolium*, this maintenance of seed banks is still dependent on long years of development, hampered by irregular flowering throughout the reproductive season [32], which implies the necessity to frequently add to existing collections. Single stranded RNA is notoriously unstable and degrades even in dry stored seeds. It was reported that the degradation of long mRNAs is stronger in aged seeds [6,33]. Other studies focused on RNA degradation including seed water content [34], RIN (RNA integrity number) [7,35], transcriptome and gene expression levels [9,22]. This makes mRNA associated processes a focal point in seed storage studies.

The molecular mechanisms behind aging of seeds are associated with oxidation of molecules such as nucleic acids, lipids and proteins, and protection from these effects with production of antioxidant, reduction of metabolism and active repair of nucleic acids [36]. In the current study, GO enrichment analysis showed that the expression control and gene silencing pathways, such as miRNAs, developmental, transcription and metabolism genes are up-regulated in all treatment comparisons. Although we found several enriched gene silencing pathways, there are several others associated with the cellular developmental process, cell differentiation, or in response to external stimulus, such as cellular response to light stimulus. Other studies also indicated that abscisic acid (*ABA*) is involved in seed dormancy and desiccation tolerance [37]. In this study, one gene glycine-rich domain-containing protein 1 (*GRP* proteins) in treatment comparison was identified as down-regulated. Increased expression levels of GRP proteins was associated with *ABA* induction in other species [38,39,40]. In both treatments, *ABC* transporter B family members are up-regulated. Other studies suggest that these genes are associated with abscisic acid [41,42], revealing the need for further investigation of these genes regarding their expression and abscisic acid content in *A. fraxinifolium*. These results suggests that this set of genes may be useful for future evaluation of seed viability in *A. fraxinifolium*. Despite the antagonistic effects of signaling pathways of ethylene and *ABA*, the presence of another down-regulated gene, Ethylene-responsive transcription factor *RAP2-12*, suggests a complex interaction, since both inhibit root growth after germination [43,44,45]. Interestingly, among genes common to both treatments, casein kinase 1-like protein 1 was suggested as a positive mediator of *ABA* signaling in *Arabidopsis* [46], but in this study it was shown to be down-regulated. When considering the other common genes, one sterol synthesis related gene (3beta-hydroxysteroid-dehydrogenase/decarboxylase isoform 1) [47] was found as down regulated. Low levels of sterol contents result in inhibition of germination, while high levels induced earlier germination [48,49]. Another down regulated gene, Protein *FAR1-RELATED*, is a component of the phytochrome A signaling pathway and found to be involved in abscisic acid (*ABA*) signaling, UV-B signaling, and reactive oxygen species (*ROS*) homeostasis, among others [50,51]. These results indicate that these pathways and differentially expressed genes can be further analyzed in the future for the development of an expression diagnostics tool for seed aging.

Of the up-regulated genes of aged seeds from Goiás (GOIAS), DEGs with the highest expression value were related to the processes of protein kinase, protein helicase, microtubule proteins, cell membrane components, polymerase and transport of carbohydrates. From the up-regulated genes of aged seeds from Minas Gerais (MINAS), the DEGs with the highest expression value were related to the processes DNA transcription, ubiquitin proteins, starch biosynthesis, transport of zinc and Late embryogenesis abundant protein (*LEA*) proteins. It has been shown that the synthesis of *LEA* proteins and heat shock proteins (HSP) is associated with longevity [52,53,54,55]. Changes were reported in gene transcript abundance of *Arabidopsis* during seed maturation and desiccation, related to regulation of *LEA* and heat-shock proteins, DNA repair, organelle protein synthesis, decrease in the metabolism of carbohydrate, amino acid and nucleic acids, sugar transport, abiotic stress, starch synthesis, synthesis of storage proteins and synthesis of hormones [10]. *LEA* proteins are synthesized at the end of seed formation and are involved in protecting the plant from damage caused by environmental stresses, especially drought, cold and salinity, and are particularly related to protecting mitochondrial membranes from dehydration damage. Heat shock proteins (*HSP*) are molecular chaperones produced by cells that oppose stress-induced denaturation of other proteins [56,57].

In a genome-wide association study (GWAS) with *A. thaliana* using transgenic plants, knockout mutants for late embryogenesis abundant (*LEA*) protein demonstrated a drastic reduction in germination after 18 months of natural aging of the seeds, as well as in artificial aging treatments. Also, a mutant for another protein related to photosystem I (PSAD1) was also reported to exhibit the same patterns of low germination [58]. Thus, the results obtained here indicate that *LEA* may be a target gene for the development of future molecular tests in *A. fraxinifolium*, as well as the common proteins among the treatments identified in GO enrichment for response to light and UV stimulus.

Other changes in metabolism that affect seed longevity are often associated with oxidative damage, such as lipid peroxidation and formation of reactive oxygen species [17,59]. Several studies have indicated the presence of a large number of proteins involved in the response to oxidative stress in dry mature seeds and in germination [60,61,62]. In addition, antioxidants such as glutathione [63], tocopherols [64] and flavonoids present in the integument [65] also play a role in longevity by relieving the oxidation that occurs during storage. In our study, antioxidant like glutathione S transferase U17 were identified among the MINAS up-regulated DEGs of aged seeds. To control cell damage caused by free radicals, seeds have developed a detoxification mechanism that includes antioxidant enzymes like catalase, ascorbate peroxidase, glutathione peroxidase, glutathione reductase, among others [66]. In addition, ubiquitin proteins were found to be down-regulated in the treatments. Ubiquitin proteins play a role in the integration of environmental stimuli and signaling pathways, which result in complex interactions in response to environmental adversities, hormone responses, plant growth and development (also seed longevity), and are involved in the protection system [67,68,69,70,71].

When considering the differences between the two accessions, for MINAS the most abundant terms for the up-regulation genes are related to DNA repair, chromatin organization, transcription regulation and protein ubiquitination. On the other hand, in the GOIAS accession, the most abundant terms are transcriptional regulation, stimulus response, and transport. DNA repair is associated with seed longevity, so this intense activity accompanied by DNA synthesis is indicative of germination, where accumulated DNA damage is repaired early in imbibition [72,73,74,75]. However, in both accessions the Gene Ontology enrichment indicates processes of gene silencing regulation. In MINAS, the most representative enrichment category is the production of miRNAs involved in gene silencing by miRNA; whereas in GOIAS Gene Ontology, enrichment showed a high fold enrichment for gene silencing. The most representative term is negative regulation of gene silencing, followed by several processes involved in the cellular development process, possibly indicating that seeds are preparing to enter cell division. This scenario reinforces the greater seed viability of the GOIAS accession as shown in Table 1 by the higher germination compared to MINAS, in which the latter is already in advanced cellular and nuclear organization in relation to germination.

The control of gene expression during development requires a set of protein complexes that act on chromatin, methylation sites, histones and as transcription factors that modulate expression. Thus, the identification of such factors/mediators associated with transcription are of great importance transcriptome studies, including on seed viability. We identified several transcription factors that have already been associated with germination and embryo development in plants. Considering the transcription factors differentially expressed in the treatments, there is a large presence of transcriptional complex mediators and transcription factor *TFIID* associated with RNA polymerase II. *TFIID* has a central role in the transcription complex of RNA polymerase [76]. The mediators are co-factors which can increase or decrease expression and are also related to signaling pathways in plants [77,78,79]. Some mediators, such as MED21 in *Arabidopsis*, are required for embryo development and cotyledon expansion [80]. In addition, some mediators are described as being related to hormones, such as brassinosteroid and abscissic acid [81]. Regarding transcription factors, we identified the transcription factor *RF2b*, a *bZIP* (basic leucine zipper) associated as a regulator of expression in response to tungro disease in rice [82]. Other studies indicate that *bZIPs* are related to seed maturation in *Arabidopsis* and peanuts [83,84]. Another one, transcription factor *UNE10*, is related to seed desiccation sensitivity in *Quercus* [85] and in the regulation of cotyledon germination in *Camellia oleifera* [86]. The transcriptional adapter *ADA2b* is related to histone modifications, as well as affecting development in *Arabidopsis* [87,88].

Present only in the comparison of GOIAS control vs. GOIAS aged-seeds, ethylene-responsive transcription factor *RAP2-12* is associated with gene expression under hypoxia in *Arabidopsis*, contributing to control oxidative stress situations, where the overexpression of this type of transcription factor increased survival of plants in mutant plants [89,90,91]. Furthermore, there is the presence of transcriptional activator *DEMETER*. These are associated with the DNA demethylase gene, acting on the plant gene imprinting and modifying the chromatin structure [92,93,94]. Another group of factors associated with germination are calmodulin binding transcription activators, reported as essential to Na+ homeostasis, hormonal signaling pathways and processes related to the development of plants [95,96,97]. In MINAS control vs. MINAS aged-seeds, the transcription factor *GTE10* (Global Transcription Factor Group E) was found, which is associated with signaling of *ABA* and sugar [98]. In GOIAS aged-seeds vs. MINAS aged-seeds comparison, the transcriptional corepressor *SEUSS* is present, associated with embryonic development in *Arabidopsis*, and its regulation of gene expression is related in stem cells [99,100].

Overall, a string of transcription factors and associated genes appear to play a role in the response to seed ageing. Some of these have already been described in seed germination or viability in other species, while others are novel in this context. Thus, the investigation of molecular mechanisms of seed longevity in this study can contribute to this and other native species. The indication of differentially expressed genes such as *LEA* and others from the photosystem, *GRP* and the ubiquitin-conjugating enzyme can serve as the basis for future investigations and contribute to the functional characterization of the seed aging process in *A. fraxinifolium*.

## 4. Materials and Methods

### 4.1. Sampling

Seed samples were obtained in 2013 from two mother trees of Cerrado biome at Goiás (coordinates 14°39′32.00′′ S and 48°35′21.00′′ W) and Minas Gerais (coordinates 16°45′38.80′′ S and 43°53′02.10′′ W) Brazilian States. After cleaning, seeds of Minas Gerais (MINAS accession) and Goiás (GOIAS accession) were stored at 18% relative humidity (RH) and 5 °C. Following a previous study of seed longevity and viability [101], these two accessions were selected based on their longevity, which were high for GOIAS and low for MINAS. Aged and control seeds were used for both accessions (Figure 1). Each accession obtained corresponds to the seeds from one tree. The seed collection was authorized for activities with a scientific purpose, under number 41166-1, from Chico Mendes Institute for Biodiversity Conservation (ICMBio), which is linked to the Ministry of the Environment (MMA).

### 4.2. Artificial Aging Treatment

Embryonic axes were extracted from control and artificially aged seed after 21 days (temperature of 45 °C and RH of 60%) according to [101]. Briefly, the viability data were transformed into probit units for sigma and P_50_ calculations [102]. Sigma indicates the time, in days, that shows a decrease of one probit unit, and P_50_ refers to the time, in days, that seeds lose 50% of viability. Germination tests were performed according to Regras para Análise de Sementes [103]. For this, four replicates of 25 seeds were placed on filter paper rolls and moistened with 2.5 times the weight in deionized distilled water, then rolled up and placed in transparent plastic bags. Seeds were placed in a BOD-type incubator at 30 °C for ten days with a photoperiod of eight hours of dark and 16 hours of light using tubular fluorescent lamps 20WT1 with a fluency rate of 30 µmol m^−2^s^−1^. Germination was scored daily and seeds were considered germinated at least 2 mm of radicle protrusion. Prior germination tests established 30 °C as the optimal germination temperature. The embryonic axes extracted from seeds without protruded radicles after 20 h imbibition were immersed in liquid nitrogen and stored at −80 °C until conducting the RNA extraction [101].

### 4.3. Library Construction and Transcriptome Sequencing

RNA extraction was done by pooling 100 embryo axes per sample. Ageing conditions used for RNA-Seq are described in the Artificial Aging Treatment section. Embryos were ground in liquid nitrogen and RNA was extracted using a NucleoSpin^®^ RNA Plant kit (Macherey-Nagel, Dürden, Germany) following the manufacturer’s instructions. RNA concentration and purity were determined using a spectrophotometer (Nanodrop-2000, Thermofisher Scientific, Waltham, MA, USA). Library construction was done using a TrueSeq RNA Library Prep Kit V2^®^ kit (Illumina, San Diego, CA, USA) and sequenced in a single lane 100 bp paired-end run in HiSeq2500 (Illumina, San Diego, CA, USA).

### 4.4. De Novo Assembly, Functional Annotation and Differential Gene Expression

Raw reads were trimmed and filtered with QV lower than 30 with Trimmomatic [104]. Transcriptome de novo assembly was done using Trinity [105]. Transcripts were annotated by Trinotate (https://trinotate.github.io/; accessed on 1 January 2022) using the Swissprot-UniProt database. Transcripts abundances were calculated by RSEM [106]. The analysis of differentially expressed genes (DEGs) was performed as follows: (1) GOIAS aged vs. control; (2) MINAS aged vs. control; (3) GOIAS vs. MINAS aged seeds; and (4) GOIAS vs. MINAS control seeds. A differential gene expression analysis was performed using DEseq [107] by the Benjamin-Hochberg adjusted *p-*value method (Padj) ≤ 0.05 as cut-off and log2fold change ≥2. Uniprot ID list from differentially expressed transcripts were used for functional analysis annotation and enzyme commission numbers (EC numbers) were assigned to differentially expressed genes according to the functional annotation data retrieved from Uniprot through Blast2GO software v. 5.2.1 (Valencia, Spain) [108]. Gene Ontology (GO) enrichment analysis was performed using the ShinyGO software [109] using *Arabidopsis thaliana* genome as a model and with a significance level of 0.05 (hypergeometric test) with False Discovery Rate (FDR) as an adjustment method. The top 20 enriched pathways were used for gene function investigation and functional category clustering.

## Figures and Tables

**Table 1:** Seed longevity upon ageing at 45 °C and at different relative humidities; and germination after 0, 8, 18 and 21 days of ageing at 45 °C and 60% RH. Sigma and P_50_ values in days.

	Relative Humidity			
		60%	65%	70%
**GOIAS**	Sigma	17.65	10.97	4.82
	P_50_	69.50	35.30	24.50
**MINAS**	Sigma	9.91	5.74	3.17
	P_50_	26.90	15.60	8.80
	**Germination (%)**			
	0 days	8 days	18 days	21 days
**GOIAS**	98	97	96	97
**MINAS**	93	90	92	81

**Table 2:** Number of reads obtained from sequencing on the Illumina HiSeq2500^®^ platform of embryonic axes of *A. fraxinifolium* seeds from treatments without and with an induced aging process.

Treatment	Total Number of Reads	Number of Trimmed Reads	Mapped Reads	% Mapped Reads	Reads not Mapped	% Reads not Mapped
GOIAS control × aged seeds	73,058,144	69,236,758	59,600,544	86.08	9,636,214	13.91
MINAS control × aged seeds	128,874,380	121,011,132	104,046,698	85.98	16,964,434	14.01
GOIAS aged × MINAS aged seeds	103,382,264	97,804,592	85,003,636	86.91	12,800,956	13.08
GOIAS control × MINAS control	95,814,580	90,062,502	76,652,780	85.11	13,409,722	14.88

**Table 3:** Selection of up- and down-regulated genes of treatments with a possible relation to the aging of *A. fraxinifolium* seeds.

	ID	Gene Ontology	Gene Function
**GOIAS up-regulated genes (GOIAS control vs. induced aged seeds)**	c20908_g1_i5	ZINC	PHD finger protein ING1 (Protein INHIBITOR OF GROWTH 1)
	c26185_g2_i3	TRANSP. MAGNESIUM	Probable magnesium transporter NIPA6
	c26616_g1_i3	ASPARTIC	Aspartic proteinase 39
	c27188_g2_i2	COMP. MEMBRANE	AP3-complex subunit beta-A
	c27812_g1_i1	MICROTUBULE	65-kDa microtubule-associated protein 6
	c28091_g1_i6	HELICASE	Pre-mRNA-splicing factor ATP-dependent RNA helicase DEAH7
	c29015_g2_i10	TRANSCRIPTION	Protein ALWAYS EARLY 3
	c29097_g1_i10	KINASE	Serine/threonine-protein kinase PBL34
	c29650_g1_i7	MEMBRANE COMPONENT	DUF21 domain-containing protein At4g14240
	c30095_g2_i20	AMINOTRANSFERASE	Branched-chain-amino-acid aminotransferase-like protein 2
	c30277_g1_i5	HELICASE	Probable helicase MAGATAMA 3
	c30355_g2_i6	DNA POLYMERASE	DNA-directed RNA polymerase III subunit 1
	c30426_g3_i2	CoA LIGASE	4-coumarate-CoA ligase-like 9
	c30654_g1_i9	KINASE	C-type lectin receptor-like tyrosine-protein kinase At1g52310
	c30712_g3_i1	SUGAR TRANSPORT	Probable sugar phosphate/phosphate translocator At1g06470
	c30834_g1_i8	NAP1	Protein NAP1
	c31008_g2_i9	TPS1	Alpha,alpha-trehalose-phosphate synthase
	c31494_g2_i12	KINASE	Probable leucine-rich repeat receptor-like protein kinase At5g49770
**MINAS up-regulated genes (MINAS control vs. induced aged seeds)**	c12237_g1_i2	CARBOHYDRATE TRANSPORT	Probable sugar phosphate/phosphate translocator At3g14410
	c18835_g1_i2	NUTRIENT RESERVE	Vicilin-like seed storage protein At2g28490
	c20908_g1_i3	ZINC TRANSPORT	PHD finger protein ING1
	c21711_g1_i3	FATTY ACID OXIDATION	Protein HOTHEAD
	c22798_g1_i3	XILAN CATABOLISM	Beta-D-xylosidase 4
	c24339_g1_i1	LIPID TRANSPORT	Late embryogen esis abundant protein D-29
	c25778_g1_i1	POLYAMINE TRANSPORT	Probable polyamine transporter At3g19553
	c27644_g1_i4	TRANSCRIPTION	Transcription factor GTE10
	c27917_g1_i3	STARCH BIOSYNTHESIS	Granule-bound starch synthase 1, chloroplastic
	c28093_g1_i3	HELICASE	ATP-dependent helicase BRM
	c28543_g1_i4	UBIQUITIN PROTEIN	Protein FIZZY-RELATED 2
	c28665_g1_i2	TOXIC SUBSTANCES CATABOLISM	Glutathione S-transferase U17
	c29854_g1_i7	NUCLEAR ORGANIZATION	Protein CROWDED NUCLEI 1
	c30020_g1_i5	ZINC TRANSPORT	Putative zinc transporter At3g08650
	c30925_g1_i2	STARCH BIOSYNTHESIS	4-alpha-glucanotransferase DPE2
	c30946_g1_i1	UBIQUITIN PROTEIN	Prob. ubiquitin-conjugating enzyme E2 24
	c30965_g1_i3	TRANSCRIPTION	Two-component response regulator-like APRR5
	c31176_g2_i7	DNA POLYMERASE II	DNA polymerase epsilon catalytic subunit A
**GOIAS down-regulated genes (GOIAS control vs. induced aged seeds)**	c20908_g1_i3	ZINC	PHD finger protein ING1
	c21777_g1_i4	ZINC	Pentatricopeptide repeat-containing protein At1g19720
	c23560_g1_i3	KINASE	Probable inactive leucine-rich repeat receptor-like protein kinase At1g66830
	c23779_g4_i5	TRANSCRIPTION	Protein FAR1-RELATED SEQUENCE 5
	c24185_g1_i5	LYSINE	4-hydroxy-tetrahydrodipicolinate synthase, chloroplastic
	c26084_g1_i5	KINASE	Casein kinase I isoform delta-like
	c26241_g6_i5	ACID NUCLEIC POLY(A)	Polyadenylate-binding protein RBP47B
	c26944_g1_i1	ATP	Feruloyl esterase A
	c27020_g1_i4	ABA	Glycine-rich domain-containing protein 1
	c27495_g7_i6	TRANSCRIPTION	Protein RTF1 homolog
	c27942_g1_i1	ACID NUCLEIC CONNECTION	Putative G3BP-like protein
	c28093_g1_i5	HELICASE	ATP-dependent helicase BRM
	c28984_g1_i3	STEROL SYNTHESIS	3beta-hydroxysteroid dehydrogenase/decarboxylase isoform 1
	c29201_g1_i2	ETHYLENE	Ethylene-responsive transcription factor RAP2-12
	c29563_g1_i8	STARCH	Phosphoglucan phosphatase LSF1, chloroplastic
	c29870_g1_i3	ATP	ABC transporter C family member 4
	c29888_g6_i1	ATP	Endoribonuclease Dicer homolog 1
	c30277_g1_i3	HELICASE	Probable helicase MAGATAMA 3
	c30555_g2_i7	RNA POLYMERASE II	Mediator of RNA polymerase II transcription subunit 23
	c30654_g1_i3	KINASE	C-type lectin receptor-like tyrosine-protein kinase At1g52310
	c30709_g2_i2	ACID NUCLEIC CONNECTION	Polyribonucleotide nucleotidyltransferase 1, chloroplastic
	c31008_g2_i6	TPS1	Alpha,alpha-trehalose-phosphate synthase
	c31157_g1_i3	UBIQUITIN PROTEIN	BTB/POZ domain-containing protein At1g04390
**MINAS down-regulated genes (MINAS control vs. induced aged seeds)**	c12237_g1_i3	CARBOHYDRATE TRANSPORT	Prob. sugar phosphate/phosphate translocator At3g14410
	c20908_g1_i5	RNA TRANSCRIPTION	PHD finger protein ING1 (Protein INHIBITOR OF GROWTH 1)
	c22798_g1_i2	XYLAN CATABOLISM	Beta-D-xylosidase 4
	c25158_g2_i2	MICROTUBULES	Tubulin alpha chain
	c26737_g1_i2	UBIQUITIN PROTEIN	E3 ubiquitin-protein ligase At4g11680
	c27190_g4_i4	UBIQUITIN PROTEIN	Protein pleiotropic regulatory locus 1
	c28984_g1_i3	STEROL BIOSYNTHESIS	3beta-hydroxysteroid dehydrogenase/decarboxylase isoform 1
	c29111_g2_i10	RNA POLYMERASE	Transcription initiation factor TFIID sub. 2
	c29114_g2_i8	AMINO ACID TRANSPORT	Cationic amino acid transporter 9, chloroplastic
	c29438_g1_i2	ROOT GROWTH	Boron transporter 1
	c29489_g1_i2	RNA TRANSCRIPTION	Protein FAR1-RELATED SEQUENCE 5
	c30224_g2_i4	CYTOSOL	F-box protein At1g78280
	c30545_g1_i5	CARBOHYDRATE CATABOLISM	Beta-galactosidase 5 (Lactase 5)
	c30986_g1_i2	DNA POLYMERASE	DNA polymerase zeta catalytic subunit
	c31176_g2_i8	DNA POLYMERASE	DNA polymerase epsilon catalytic sub. A

**Table 4:** Transcription-related factors identified as differentially expressed in the different treatments in this study of *A. fraxinifolium*.

GOIAS Control vs. GOIAS Aged-Seeds		
id	sprot_Top_BLASTX_hit	Description
c30555_g2_i9	MED23_ARATH	Mediator of RNA polymerase II transcription subunit 23
c29094_g5_i2	CMTA4_ARATH	Calmodulin-binding transcription activator 4
c29111_g2_i6	TAF2_ARATH	Transcription initiation factor TFIID subunit 2
c22254_g1_i5	RF2B_ORYSJ	Transcription factor RF2b
c29294_g1_i2	MED12_ARATH	Mediator of RNA polymerase II transcription subunit 12
c27419_g2_i1	TAD2B_ARATH	Transcriptional adapter ADA2b
c24172_g1_i1	UNE10_ARATH	Transcription factor UNE10
c31090_g2_i9	MD37D_ARATH	Probable mediator of RNA polymerase II transcription subunit 37c
c29201_g1_i2	RA212_ARATH	Ethylene-responsive transcription factor RAP2-12
c30555_g2_i7	MED23_ARATH	Mediator of RNA polymerase II transcription subunit 23
c29246_g1_i1	DME_ARATH	Transcriptional activator DEMETER
c24653_g1_i4	KELP_ARATH	RNA polymerase II transcriptional coactivator KELP
c26529_g2_i3	MD33A_ARATH	Mediator of RNA polymerase II transcription subunit 33A
**MINAS Control vs. MINAS Aged-Seeds**		
**id**	**sprot_Top_BLASTX_hit**	**Description**
c27644_g1_i4	GTE10_ARATH	Transcription factor GTE10
c29111_g2_i10	TAF2_ARATH	Transcription initiation factor TFIID subunit 2
**GOIAS Aged-Seeds vs. MINAS Aged-Seeds**		
**id**	**sprot_Top_BLASTP_hit**	**Description**
c29111_g2_i10	TAF2_ARATH	Transcription initiation factor TFIID subunit 2
c29413_g3_i6	PUR_ARATH	Transcription factor Pur-alpha 1
c30283_g2_i3	SEUSS_ARATH	Transcriptional corepressor SEUSS
c30495_g3_i3	UNE12_ARATH	Transcription factor UNE12
c30555_g2_i6	MED23_ARATH	Mediator of RNA polymerase II transcription subunit 23

## Data Availability

The data presented in this study are openly available in Genbank, accession numbers under project PRJNA881610.

## Supplementary Materials

The supporting information can be downloaded at: https://www.mdpi.com/article/10.3390/ijms232213852/s1.
