# Supplementary material for: Comparative Seeds Storage Transcriptome Analysis of Astronium fraxinifolium Schott, a Threatened Tree Species from Brazil
Source: Int J Mol Sci. 2022 Nov 10;23(22):13852. doi: 10.3390/ijms232213852 (PMC9696909; doi:10.3390/ijms232213852)
Supplement: Supplementary file 1 [file ijms-23-13852-s001.zip › Supplemental table S2.pdf]

Table S2. Common genes identified between artificially aged seeds of GOIAS and MINAS treatments.

	Transcript ID	Uniprot/SwissProt ID	Protein names
**up-regulated common genes**	c31142_g1_i6	AB26B_ARATH	ABC transporter B family member 26, chloroplastic (ABC transporter ABCB.26) (AtABCB26) (Antigen peptide transporter-like 1) (Transporter associated with antigen processing-like protein 1) (AtTAP1)
	c29015_g2_i10	MYO15_ARATH	Myosin-15 (Myosin XI I) (AtXI-I)
**down-regulated common genes**	c26084_g1_i2	KC1D_ARATH	Casein kinase 1-like protein 1 (EC 2.7.11.1) (Casein kinase 1)
	c28984_g1_i3	HSDD1_ARATH	3beta-hydroxysteroid-dehydrogenase/decarboxylase isoform 1 (At3BETAHSD/D1) (EC 1.1.1.418
	c29489_g1_i2	FRS5_ARATH	Protein FAR1-RELATED SEQUENCE 5
	c27880_g2_i1	M030_ARATH	Uncharacterized mitochondrial protein AtMg00030 (ORF107a)
	c31473_g3_i2	-	no BLAST results/Uncharacterized protein
	c20285_g1_i1	-	no BLAST results/Uncharacterized protein
	c20285_g2_i1	-	no BLAST results/Uncharacterized protein
	c20285_g1_i3	-	no BLAST results/Uncharacterized protein
	c15514_g1_i1	-	no BLAST results/Uncharacterized protein
