# Supplementary material for: Comparative Seeds Storage Transcriptome Analysis of Astronium fraxinifolium Schott, a Threatened Tree Species from Brazil
Source: Int J Mol Sci. 2022 Nov 10;23(22):13852. doi: 10.3390/ijms232213852 (PMC9696909; doi:10.3390/ijms232213852)
Supplement: Supplementary file 1 [file ijms-23-13852-s001.zip › Supplemental table S3.pdf]

Table S3. Gene Ontology (GO) enrichment analysis of DEGs that were up and down expressed in the comparison of GOIAS control vs GOIAS aged-seeds.

**GOIAS control vs GOIAS aged-seeds up-regulated genes**				
**Enrichment FDR**	nGenes	Pathway Genes	Fold Enrichment	Pathway
**0.000317**	3	9	88.55128	Negative regulation of gene silencing
**6.70E-05**	6	70	22.77033	Trichome morphogenesis
**0.000292**	5	60	22.13782	Negative regulation of gene expression, epigenetic
**0.000158**	6	90	17.71026	Trichome differentiation
**0.000222**	6	100	15.93923	Plant epidermis morphogenesis
**0.000367**	7	183	10.16162	Gene silencing
**0.000367**	8	258	8.237329	Cell morphogenesis involved in differentiation
**0.000116**	10	342	7.767656	Negative regulation of gene expression
**0.000317**	9	329	7.267126	Chromatin organization
**0.000116**	11	423	6.908256	Cell morphogenesis
**0.000397**	9	349	6.850672	Cell development
**4.30E-05**	16	841	5.054057	Cellular developmental process
**0.000418**	12	665	4.793754	Transcription by RNA polymerase II
**0.000317**	13	737	4.685889	Negative regulation of macromolecule metabolic proc.
**0.000367**	13	759	4.550066	Cell differentiation
**0.000158**	16	1015	4.187647	Anatomical structure morphogenesis
**4.30E-05**	19	1217	4.147431	Negative regulation of biological proc.
**4.30E-05**	24	1845	3.45566	Organelle organization
**0.000222**	20	1606	3.308267	Post-embryonic development
**GOIAS control vs GOIAS aged-seeds down-regulated genes**				
**Enrichment FDR**	nGenes	Pathway Genes	Fold Enrichment	Pathway
**0.02139**	4	86	15.2979	Regulation of gene expression, epigenetic
**0.019264**	5	143	11.50017	RNA catabolic process
**0.036112**	4	119	11.05562	mRNA catabolic process
**0.026204**	5	185	8.889318	Nucleobase-containing compound catabolic proc.
**0.019264**	6	229	8.617592	Vegetative to reproductive phase transition of meristem
**0.021436**	6	266	7.418904	Aromatic compound catabolic proc.
**0.022452**	6	275	7.176104	Organic cyclic compound catabolic proc.
**0.045518**	5	235	6.997974	Heterocycle catabolic proc.
**0.045518**	5	237	6.938919	Cellular nitrogen compound catabolic proc.
**0.019264**	7	342	6.731969	Negative regulation of gene expression
**0.036112**	6	329	5.998263	Chromatin organization
**0.036112**	8	605	4.349154	mRNA metabolic process
**0.036112**	9	737	4.016476	Negative regulation of macromolecule metabolic proc.
**0.019264**	13	1217	3.513362	Negative regulation of biological proc.
**0.020374**	13	1265	3.380049	Cellular response to stress
