# Supplementary material for: Comparative Seeds Storage Transcriptome Analysis of Astronium fraxinifolium Schott, a Threatened Tree Species from Brazil
Source: Int J Mol Sci. 2022 Nov 10;23(22):13852. doi: 10.3390/ijms232213852 (PMC9696909; doi:10.3390/ijms232213852)
Supplement: Supplementary file 1 [file ijms-23-13852-s001.zip › Supplemental table S4.pdf]

Table S4. Gene Ontology (GO) enrichment analysis of DEGs that were up expressed in the comparison of MINAS control vs MINAS aged-seeds.

**MINAS control vs MINAS aged-seeds up-regulated genes**				
**Enrichment FDR**	nGenes	Pathway Genes	Fold Enrichment	Pathway
**0.042939**	2	28	73.08995	Production of miRNAs involved in gene silencing by miRNA
**0.035833**	3	104	29.51709	Negative regulation of post-embryonic development
**0.040157**	3	124	24.75627	Negative regulation of multicellular organismal process
**0.042939**	3	151	20.32965	Negative regulation of developmental process
**0.000699**	9	1217	7.567242	Negative regulation of biological process
