# Supplementary material for: Comparative Seeds Storage Transcriptome Analysis of Astronium fraxinifolium Schott, a Threatened Tree Species from Brazil
Source: Int J Mol Sci. 2022 Nov 10;23(22):13852. doi: 10.3390/ijms232213852 (PMC9696909; doi:10.3390/ijms232213852)
Supplement: Supplementary file 1 [file ijms-23-13852-s001.zip › Supplemental table S5.pdf]

Table S5. Gene Ontology (GO) enrichment analysis of DEGs that were up and down expressed in the comparison of GOIAS aged-seeds vs MINAS aged-seeds.

**GOIAS aged-seeds vs MINAS aged-seeds up-regulated genes**				
**Enrichment FDR**	nGenes	Pathway Genes	Fold Enrichment	Pathway
**8.03E-05**	3	4	259.0125	Cellular response to UV-C
**0.010182**	2	8	86.3375	Transcription, RNA-templated
**0.00094**	3	13	79.69615	Response to UV-C
**0.002755**	4	60	23.02333	Negative regulation of gene expression, epigenetic
**0.002755**	4	62	22.28065	Detection of external stimulus
**0.000895**	5	86	20.07849	Regulation of gene expression, epigenetic
**0.000895**	6	137	15.12482	Cellular response to light stimulus
**0.000895**	6	144	14.38958	Cellular response to radiation
**0.009287**	4	97	14.24124	Phosphatidylinositol metabolic process
**0.009287**	5	183	9.435792	Gene silencing
**0.000895**	8	329	8.397568	Chromatin organization
**0.002894**	9	564	5.510904	Chromosome organization
**0.007264**	10	813	4.247847	Cellular lipid metabolic process
**0.009287**	12	1217	3.405259	Negative regulation of biological process
**0.010182**	15	1845	2.807724	Organelle organization

**GOIAS aged-seeds vs MINAS aged-seeds down-regulated genes**				
**Enrichment FDR**	nGenes	Pathway Genes	Fold Enrichment	Pathway
**0.023395**	9	850	5.318759	Organonitrogen compound catabolic process
**0.023395**	11	1422	3.885795	Cellular catabolic process
**0.023395**	13	1914	3.411836	Response to external stimulus
